# Early screening the small bowel is key to protect Peutz-Jeghers syndrome patients from surgery: a novel mutation c.243delG in *STK11* gene

**DOI:** 10.1186/s12876-019-0987-z

**Published:** 2019-05-09

**Authors:** Yu-Liang Jiang, Zi-Ye Zhao, Bai-Rong Li, Jing Li, Xiao-Wei Jin, En-Da Yu, Xiao-Dong Xu, Shou-Bin Ning

**Affiliations:** 1 Department of Gastroenterology, Beijing Shijitan Hospital, 10 Tieyi Rd., Beijing, 100038 China; 2 Department of Gastroenterology, Airforce Medical Center of PLA, 30 Fucheng Rd., Beijing, 100142 China; 3 Department of Colorectal Surgery, Shanghai Changhai Hospital, 168 Changhai Rd., Shanghai, 200433 China

**Keywords:** Peutz-Jeghers syndrome, *STK11* gene, De-novo mutation, Hamartoma, Polyposis

## Abstract

**Background:**

Peutz-Jeghers syndrome (PJS) is a Mendelian disease, whose causative gene is *STK11*, mainly characterized by gastrointestinal polyposis and increased cancer risk. Clinical observation reveals intussusception in childhood are more frequent and severe than in adults, and it is difficult to prevent this knotty complication.

**Case presentation:**

A boy without a positive family history grew oral MP after birth and developed abdominal pain and bloody stood at 7 years old. Endoscopy revealed multiple polyps within the colon and the ileum, and endoscopic polypectomy and regular surveillance protected him from severe complications and open surgeries. A heterozygous deletion in *STK11*, c.243delG, was detected in the proband but not in his parents. This mutation has not been documented in databases.

**Conclusions:**

We suspect a child of PJS may need a more thorough endoscopic examination including enteroscopy or capsule endoscopy to take care of small bowel when PJS related symptoms comes up.

## Background

Peutz-Jeghers Syndrome (PJS, OMIM 175200), an autosomal dominant disorder, is characterized by three major clinical features, which are mucocutaneous pigmentation (MP), gastrointestinal (GI) hamartomatous polyps and an increased risk for GI and extra-GI malignancies [1]. Germline mutations in *STK11* gene are believed to be the causative reason of this syndrome, and hundreds of them recognized in PJS patients have been documented (http://www.hgmd.cf.ac.uk).

Among recorded PJS cases, more than 30% of them develop polyp-related manifestations by age 10 years and 50% by 20 years [2]. In children with PJS, intussusception due to GI polyps is the most critical symptom, while in adults, the dominant threaten are PJS-associated tumors [3]. van Lier et al. [4] described cumulative intussusception risk in PJS of 15% by age 10 years and 50% by age 20 years. Because of these circumstances, making a set of strategies for screening and surveillance is important to young patients with PJS. Hinds et al. [5] reported the natural history of PJS in the St. Mark’s Hospital Polyposis Registry and showed 68% (23/34) of the adults with PJS had undergone a laparotomy for intestinal obstruction/ intussusception before 18 years old.

Enteroscopy is the promising intervention measure for PJS, and many surgeries have been avoided since enteroscopy came into use. We report here a 9 year-old PJS boy with a novel *STK11* mutation (c.243delG) characterized by MP and GI polyps, and he has not undergone any surgical intervention due to the early use of enteroscopy. Based on his process of diagnosis and treatment, we highlight the importance of early screening for small bowel polyps.

## Case presentation

### Case study

The 9 year-old boy from Southeast China came to Airforce Medical Center of PLA early this year, who was diagnosed of PJS in a local hospital. Multiple MPs on the lips and cheeks were observed by his families shortly after his birth, and this phenomenon did not draw their attention since this family did not have a history of PJS. At the age of seven, the boy got paroxysmal abdominal cramps after meal and fresh blood came out with stool. He was soon sent to the local hospital, and colonoscopy revealed multiple colon polyps. Then endoscopic polypectomy was performed, and pathological exam confirmed them hamartomas. The symptoms relieved largely after colonoscopy. Taking pigmentation and GI hamartomas together, the diagnosis of PJS was confirmed.

**Fig. 1:**
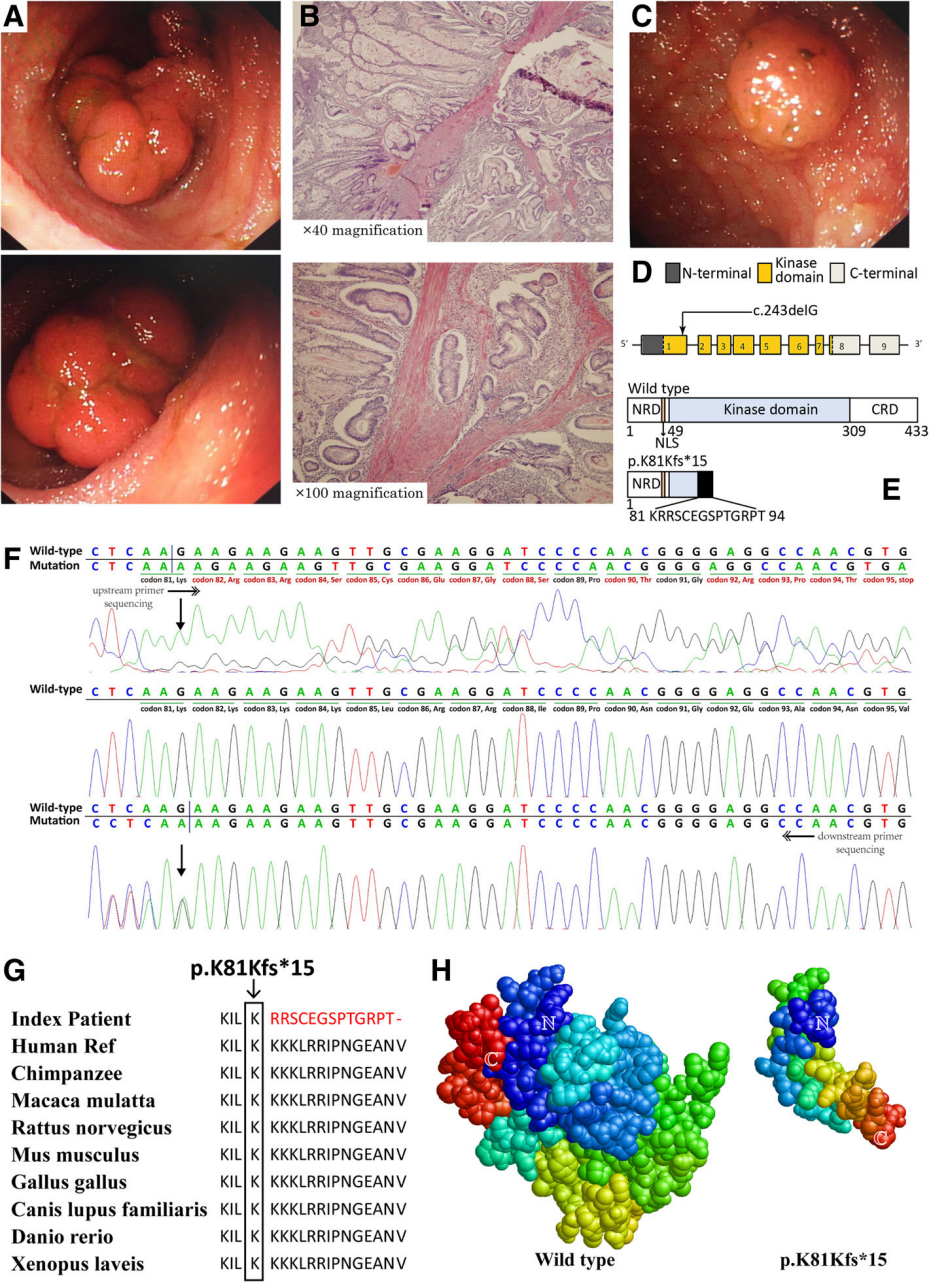
**a** Endoscopic view of the larger polyp in the ileum. **b** Representative hematoxylin-eosin-stained tissue slices of the larger ileal polyp specimens confirms hamartomatous. Up, × 40 magnification; low, × 100 magnification. **c** Endoscopic view of the smaller polyp in the ileum. **d** The structure of *STK11* gene. This novel mutation is within exon 1. **e** Schematics of the secondary structure or functional domains of the STK11 protein. The mutant protein results in a large-scale loss of kinase domain and a complete loss of the C-terminal domain compared to the wild type. NLS, Nuclear localization signal, NRD or CRD, N- or C-terminal regulatory domain. **f** Sanger sequencing forward and backward revealed a heterozygous deletion, c.243delG. **g** Evolutionary conservation of amino acid residues altered by c.243delG (p.K81Kfs*15) across different species. **h** Predicted by Swiss-Model online software, the mutant protein turns into an abnormal shape with loss of main functional domain compared with the wild type

One year later, the similar symptoms appeared again, and this time doctors in the local hospital used capsule endoscopy, during which a large polyp with the diameter of 5 cm in the ileum was detected. After expectant treatment, the patient was referred to our department for further treatment. Physical examination confirmed the MPs and revealed no other PJS related findings (testicular tumors included). We arranged enteroscopy for him after his admission, and the large polyp whose diameter was actually 2.5 cm together with another smaller one within the ileum was resected successfully (Fig. 1a and c). Postoperational pathology reported the PJS-related hamartomas which showed the classical arborizing smooth muscle, consistently with previous results (Fig. 1b).

### Mutation analysis

During the boy’s remedy, we collected the blood samples of his and his parents after their signing informed consents forms (ICF). In the laboratory, the genomic DNA was extracted from their peripheral blood leucocytes using animal genomic DNA kit (TSP201, TsingKe Biotech). Polymerase chain reactions (PCR) were performed by using modified DNA polymerase mix (TSE004, TsingKe Biotech), and the coding sequence and boundaries of STK11 gene were analyzed by DNA sequencing essentially as previously described [6, 7].

Fifty unrelated control individuals who came to our department for gastric polyps treatment were also screened for the presence of the mutation to rule out polymorphisms. All biological samples were collected after written ICF being acquired.

Sanger sequencing of *STK11* gene revealed a heterozygous germline deletion c.243delG in exon 1 in the patient’s genomic DNA (Fig. 1d and f), while it was not detected in the parents’ or the control samples’. This mutation has not been documented in literatures or mutation databases such as dbSNP, OMIM, ClinVar, HGMD or ExAC (http://exac.broadinstitute.org/). It generates a translational frameshift and a premature stop (p.K81Kfs*15), resulting in a large-scale loss of kinase domain and total loss of the C-terminal domain in comparison to the wild type (Fig. 1e).

### Structure prediction of the mutant protein and analysis of evolutionary conservation of amino acid residues

Swiss-Model online software (http://swissmodel.expasy.org), a homology modeling program [8], showed the simulated mutant protein turns into an aberrant shape with significantly smaller size (Fig. 1h). Evolutionary conservation was analyzed by comparing across different species (https://www.ncbi.nlm.nih.gov/protein/STK11), and the results showed these missing amino acid residues were most conserved (Fig. 1g).

Generally, this mutation is believed to be a novel pathogenic one in *STK11* leading to PJS in line with American College of Medical Genetics and Genomics (ACMG) classification system (Table 1).

**Table 1:** Classification of multiple evidences about the novel mutation

Evidences	c.243delG
Population data	Absent in 50 controls and population databases (PM2)
Computational and predictive data	Predicted null variant (frameshift mutation included) in *STK11* where LOF is a mechanism of PJS (PVS1)
Functional data	Not applicable
Segregation data	Cosegregation with PJS (PP1)
De novo data	De novo (without paternity & maternity confirmed) (PM6)
Conclusion	Pathogenic (1 PVS1 and 2 PM and 1 PP)

## Discussion and conclusions

Here we displaced a novel mutation in *STK11* causing PJS in a 9 year-old boy without a positive family history. The mutation was neither documented nor identified in unrelated control individuals. The fraction of de novo mutations (sporadic cases) is 49.21% (62/126) based on our database, and 63.0% (29/46) in our recent published cohort [9]. Since evolutionary conservation analysis together with structure prediction show the pathological effect of it, we are sure of that this mutation is a causative one.

In 1895, Dr. Connor first reported this syndrome in the literature, and Dr. Bruwer finally named it in 1954 after two investigators who made great contributions to it [10]. The pathogenic gene was finally believed as *STK11* and cloned in 1997 [11]. *STK11* encodes a 433-amino-acid-residue protein and it acts as a tumor suppressor. STK11 protein comes into play by participating in several pathways. For example, we noticed that impaired P53 activity caused by *STK11* mutations in PJS patients is significantly related to cancer risk [12]. Multiplex ligation–dependent probe amplification (MLPA) together with direct sequencing raise the mutation detection rate to over 60% in most instances [13]. Though frameshift and nonsense mutations are most common types, large deletions and missense mutations are also disease-causing. Interestingly, we have found significantly more splice errors in Chinese PJS patients [9]. Among reported *STK11* mutations (HGMD), most variants are in the region of catalytic kinase domain (amino acids 49–309) which cause the loss of kinase activity [14]. Through sequence alignment, the mutant protein loses main functional domains, so the novel mutation is probably a pathogenic one and the causative one to this boy, which broadens the spectrum of pathological *STK11* gene mutations.

For children without a family history, PJS is often diagnosed when they suffer from intestinal obstruction and comes to the surgical emergency [15]. Under the circumstances, it is hard to implement preventive measures to avoid a open surgery. But when the possibility of PJS is predicted based on family history or typical MPs, a screening test for high risk individuals is necessary. According to the recommendations for management 2010, baseline colonoscopy and upper GI endoscopy should be performed at the age of 8 (Table 2), and extra-intestinal part needed screening for tumors include pancreas, breast, ovary, endometrium, cervix, testis and lung [2]. This is the current and comprehensive recommendations for PJS screening, surveillance and PJS-related cancer prevention. However, we notice the baseline age is worthy of discussion. Goldstein et al. [16] reviewed the charts of 14 children with a diagnosis of PJS at Children’s Hospital Colorado from 2000 to 2011, and 5 children suffered from intussusception events at the median age of 5 years for the first screening test. They suggested revised guidelines should consider initial screening at age 4 to 5 with capsule endoscopy and upper and lower endoscopy. The onset age in this case also supports the suggestion to advance the screening baseline. While it’s not convincing enough to revise the current recommendations merely based on these clues, and more evidences are needed to address this issue clearly. For endoscopic screening in children, it’s hard to keep the balance between advantages and disadvantages. Though we find early screening may reduce PJS complications like bowel obstruction, there are many potential side effects due to technical difficulty and anesthesia risk.

**Table 2:** Current surveillance recommendations for GI tumors in PJS patients

Methods	Start time or duration
Baseline endoscopy^a^	8 years old
Polyps detected	continue 3 yearly until 50 years
No polyps detected,	repeat at 18 years, then 3 yearly until 50 years
Colonoscopy	1–2 yearly after age 50 years
Capsule endoscopy	3 yearly from age 8 years

^a^Upper GI endoscopy, colonoscopy and capsule endoscopy are all included

Once GI polyps were detected before they cause severe GI complications, enteroscopy is the effective method to avoid the open surgery [17]. Belsha et al. [18] set and followed up a prospective cohort for 6 years to examine the effectiveness of double-balloon enteroscopy (DBE) polypectomy for children with PJS. After 22 DBEs for 16 pediatric PJS patients, large polyps (>/=1 cm) were confirmed in 14 of them. Successful polypectomy were achieved by DBE or laparoscopically assisted DBE (Lap-DBE) in all patients. Besides only one patient suffered from post-Lap-DBE pelvic abscess, all other patients remained. Intervention free during follow-up (median 26 months). By analyzing the data of own 11 years’ experience, we notice DBE is effective for intestinal polyps management, and we have finished 153 DBEs in 53 pediatric patients. And when compared with DBE in adults, the incidence of perforation was higher in children (5/53 vs 4/150, *P* < 0.05) [19]. We believe with the progress of the equipment and technique, updating information is still needed to bring new recognitions. In the case reported here, ileal polyp was detected only when the symptoms happened again. If DBE were available, this patient might receive GI examination thoroughly when symptoms occurred firstly, and regular follow-up would surely protect him from severe GI complications. In our experience, within 131 PJS patients who had abdominal surgeries for intestinal obstruction before, 113 patients (86.3%) avoid a second open surgery after receiving regular follow-up by DBE [20].

## Abbreviations

DBE: Double-balloon enteroscopy
dbSNP: Database of single nucleotide polymorphisms
GI: Gastrointestinal
HGMD: Human Gene Mutation Database
ICF: Informed consents form
MLPA: Multiplex ligation–dependent probe amplification
MP: Mucocutaneous pigmentation
OMIM: Online Mendelian Inheritance in Man
PCR: Polymerase chain reactions
PJS: Peutz-Jeghers syndrome
*STK11*: Serine/threonine kinase 11
